# Removing opportunities to calculate improves students’ performance on subsequent word problems

**DOI:** 10.1186/s41235-019-0175-2

**Published:** 2019-07-16

**Authors:** Karen B. Givvin, Veronika Moroz, William Loftus, James W. Stigler

**Affiliations:** UCLA Department of Psychology, University of California Los Angeles, 1285 Franz Hall, Box 951563, Los Angeles, CA 90095-1563 USA

**Keywords:** Developmental mathematics, Community college, Relational thinking, Anxiety, Intervention

## Abstract

**Background:**

In two studies we investigated whether removing opportunities to calculate could improve students’ subsequent ability to solve similar word problems. Students were first asked to write explanations for three word-problems that they thought would help another student understand the problems. Half of the participants explained typical word problems (i.e., problems with enough information to make calculating an answer possible), while the other half explained the same problems with numbers removed, making calculating an answer impossible. We hypothesized that removing opportunities to calculate would induce students to think relationally about the word problems, which would result in higher levels of performance on subsequent transfer problems.

**Results:**

In both studies, participants who explained the non-calculable problems performed significantly better on the transfer test than participants who explained the typical (i.e., calculable) problems. This was so in spite of the manipulation not fully suppressing students’ desire to calculate. Many students in the non-calculable group explicitly stated that they needed numbers in order to answer the question or made up numbers with which to calculate. There was a significant, positive relationship between the frequency with which students made up numbers and their self-reported mathematics anxiety.

**Conclusions:**

We hypothesized that the mechanism at play was a reduction in instrumental thinking (and an increase in relational thinking). Interventions designed to help students remediate prior mathematical failure should perhaps focus less on the specific skills students are lacking, and more on the dispositions they bring to the task of “doing mathematics.”

## Significance

Large numbers of students enter community college unprepared for college-level mathematics. Their time in remedial courses delays (and sometimes thwarts) their progress toward earning a degree and the costs to both the students and the institutions that serve them is high. Interventions of different forms have been attempted with these students, from providing study skills to providing supplemental instruction; from speeding up course progressions to slowing them down; from instituting peer supports to putting materials online. In some cases, developmental (i.e., remedial) mathematics has been done away with entirely, with students instead getting “just in time” supports within the context of college credit-bearing classes. In very few cases have interventions focused on changing teaching routines. The present study tests a brief intervention that might easily translate into a routine that could be regularly implemented in classrooms. We have targeted college students with particular difficulties in mathematics because the persistent nature of their struggles suggests that innovative interventions might be called for. However, we have no reason to believe that similar routines could not be used effectively with younger students who struggle with math or even with students who do not.

## Background

Perhaps because of the way mathematics is taught in the USA (Stigler & Hiebert, 1999), many American students come to view mathematics as a set of facts and procedures to be memorized rather than as a coherent set of concepts and tools for making sense of the world (Erlwanger, 1973; Garofalo, 1989). Although parts of mathematics do require memorization, if that is the only goal students have, and if they fail to connect facts and procedures to underlying mathematical concepts, the knowledge that results is likely to be fragmented, rigid, and susceptible to bugs (Givvin, Stigler, & Thompson, 2011; Stigler, Givvin, & Thompson, 2010).

These two conceptualizations of mathematics and what it means to engage in mathematical tasks - that is, as procedures to be memorized versus tools for making sense - are consistent with what Skemp (1976) referred to as instrumental understanding (i.e., rules without reasons) and relational understanding (knowing what to do and why). He theorized that there are three compelling benefits to instrumental understanding: it is usually easier to understand, the rewards are immediate and more apparent, and it usually leads to finding an answer more quickly. However, what it lacks - and what relational understanding offers - is adaptability to novel tasks. Though relational understanding is harder to achieve, it lasts longer and provides more intrinsic satisfaction. Relational schemas “seem to act as an agent of their own growth” (p. 24), causing learners to seek out new areas to which the schemas apply, and lead to an ever-growing network of connected ideas.

The two studies reported here ask whether it is possible, through a brief intervention, to move students - even if temporarily - from an instrumental to a relational approach to solving word problems, and whether evidence of this shift might be seen in students’ success on an immediate transfer task. In testing this approach, we chose to work with a group of community college^1^ students.

Despite the fact that most community college students have passed high school algebra and should, therefore, be ready for college-level mathematics, the majority, apparently, are not. In one study, Bailey and colleagues (Bailey, Jeong, & Cho, 2010) found that 59% of students entering community colleges nationwide were placed into developmental (remedial) mathematics courses: 24% were placed one level below college entry-level mathematics (i.e., intermediate algebra), 16% two levels below (i.e., elementary algebra), and 19% three or more levels below (i.e., pre-algebra or arithmetic). What is more, only 20% of those referred to developmental mathematics successfully completed the prerequisites for college-level mathematics within 3 years of their initial placement.

In a series of interviews with community college developmental mathematics students we found ample evidence that these students bring with them instrumental views of what it means to know mathematics and what it takes to learn mathematics (Givvin et al., 2011; Stigler et al., 2010). Seventy-seven percent of students in our study indicated that knowing mathematics was simply a matter of remembering the rules and procedures. “Mathematics is just all these steps,” one of the students said. Or as another student said: “In mathematics, sometimes you have to just accept that that’s the way it is and there’s no reason behind it.” And, yet another student: “I don’t think [being good at mathematics] has anything to do with reasoning. It’s all memorization” (Givvin et al., 2011, p. 7). When, in our interviews, we asked students to answer non-standard questions that required some conceptual thinking, they usually tried to apply procedures they remembered from their past, even when no procedures were required to answer the question. They often applied these procedures inappropriately or incorrectly.

These findings are consistent with many other studies in younger students over a number of years (e.g., Erlwanger, 1973; Verschaffel & De Corte, 1997). Of particular interest is work by Stacey and MacGregor (1999), who identified a phenomenon they referred to as the “compulsion to calculate.” They found that when students are presented with a mathematics word problem, their first response often is to try to compute an answer, even before they have tried to understand the problem. The description offered by Stacey and MacGregor (1999) reminds us of the community college students we interviewed, who appeared not to think long about the problem posed, but instead to search their memory for a procedure that some teacher, at some point, had told them to use.

This phenomenon is striking because it is in such contrast to the way successful problem solvers approach problems across a number of domains. Studies of expertise have shown that experts attend more to the underlying structure of a problem, whereas novices rely more on surface features (Chi, Glaser, & Rees, 1982; Hinsley, Hayes, & Simon, 1977; Larkin, McDermott, Simon, & Simon, 1980; Larkin & Simon, 1987; Lesgold, 1988; Mayer, 1985; Newell & Simon, 1972). Research also has shown that expert problem solvers typically spend more time thinking about problems and trying to understand them than do novices, who tend to immediately execute a solution (Hegarty, Mayer, & Monk, 1995; Lesgold, 1988). That is, experts take a more relational approach, whereas the approach taken by novices is more instrumental.

Borrowing from Skemp’s theory, students’ compulsion to calculate is merely one symptom of an instrumental view of mathematics. What looks like a compulsion is simply students acting upon what they believe to be the primary requirement of the domain. Indeed, these students may be entirely unclear about what alternative is available if calculation is not.

It is possible that community college students jump to procedures because they lack the ability to think about the problem in a productive, relational way. But we do not believe this is the case. In our interviews we find evidence that these students are capable of relational thinking when supported by the prodding of a skilled interviewer (Givvin et al., 2011), and further, that when they bring that thinking to bear on problems, they also are able to produce correct answers (Stigler et al., 2010). Instead, we hypothesize that students fail to think about mathematics relationally not because they cannot, but because they do not know they are supposed to, and do not believe that doing so will help them get correct answers.

It was this line of reasoning that prompted our attempt to nudge students into thinking relationally about simple word problems (see Damgaard & Nielsen, 2018, for a review of nudging in education). We did so by preventing them from doing what they would normally do in such situations, which is to immediately calculate. Our goal was not to build a curriculum around this approach, but rather to first gather evidence to support the idea. We altered standard word problems in two ways in the two studies reported below. First, for half of the students, we removed the numbers with which they might calculate, rendering the problems non-calculable. Second, in order to create a reason for the students with non-calculable problems to engage with them (yet have consistent instructions for all students), instead of asking students to *solve* the problems, we asked them to *explain* them in a way they thought would be helpful to other students. Self-explanation - having students explain to themselves the material they are learning while they are studying it - has been shown to lead to enhanced learning and effective problem-solving (Bielaczyc, Pirolli, & Brown, 1995; Chi, de Leeuw, Chiu, & LaVancher, 1994; Fonseca & Chi, 2011; Renkl, Stark, Gruber, & Mandl, 1998; Wong, Lawson, & Keeves, 2002).

We predicted that participants forced to interact with problems for which they could not immediately engage in calculation would be more likely to develop relational representations of these problems. Instead of leaping to write down solution steps, perhaps they would grapple with (and express in writing) how quantities were related to each other, in context. We hypothesized further that these participants, because they had been nudged to think more relationally, might be slightly more able to apply what they learned in the explanation phase of the study to the solving of future (transfer test) problems.

## Study 1

### Method

#### Participants

Thirty-two students enrolled in an Introductory Algebra (developmental mathematics) course at a Southern California community college participated in the study. Although demographics for the sample were not collected, we know that the student body at the college at the time was 53% female; 59% under the age of 25 years; 55% Hispanic, 19% Asian, 11% white non-Hispanic, 4% Pacific Islander/Filipino, 3% African American, 3% multi-ethnic, and 6% other or unknown, according to the college website. Students were offered extra credit by their instructor in exchange for their participation in the study and no student in the class declined to participate. No power analysis was conducted. For this initial exploration, we simply selected a single, intact classroom.

#### Explanation phase

In the initial, explanation phase of the study, participants were presented with three mathematics problems and asked to write an explanation of each problem that they thought would “help another student understand the problem.” We used pre-algebra “part-whole” problems in which numeric values can be easily substituted with either letter variables or approximate quantities such as “some” and “more.” Because these kinds of problems can be represented in a variety of ways (e.g., drawings, literal formulas) we believed they had the potential to elicit a rich variety of explanations.

Two versions of the materials were prepared, with students randomly assigned to one of the two versions. In the calculable condition, the problems were similar to those found in standard pre-algebra textbooks. In the non-calculable condition, the same problems were presented, but problem-relevant numbers were replaced with approximate quantities, thus making it impossible to calculate an exact answer. In both conditions, students were asked only to explain the problems, not solve them. All six problems - three in each condition - are in the Appendix.

It should be noted that numbers were not removed entirely from non-calculable problems. Rather, enough of the numbers were removed from the problems to make it impossible for students to calculate a numeric answer. It might be argued that the resulting problems sound artificial. To that point we suggest that they sound no more artificial than many other problems common in mathematics texts. What sets the non-calculable problems apart is that they defy our expectations, as people familiar with standard mathematics problems, that all mathematics problems can yield numerical answers. Beyond this, it is important to keep in mind that performance on these problems, to whatever degree they were artificial, was not the focus of our study. Instead, we were investigating how *explaining* these first three problems would influence students’ ability to *solve* the standard problems that followed (on the transfer test).

#### Transfer test

After explaining three problems, students were asked to solve four transfer problems (see Appendix for specific problems). Three were similar in format and complexity to those the students had been asked to explain earlier in the study. The fourth problem was a far transfer task. That is, it was structurally different from the items in the explanation phase. It was, however, similar in complexity to the prior items. All transfer problems included numbers to render them solvable by calculation. Both experimental groups were given the same transfer test.

#### Procedure

At the beginning of a regular class period, an experimenter distributed a multi-page packet to students, randomly assigning half of the students to each experimental condition. The packet included a consent form, the three problems for the explain phase of the study (either the calculable or non-calculable form, depending on condition), and the four-problem transfer test. We intentionally kept both the experimental task and the transfer test short so that they could be administered within a normal class period, infringing as little as possible on instructional time. And rather than conduct a content knowledge pretest, we relied on random assignment to produce groups of similar incoming ability. Students were told that the aim of the study was to investigate how explanations from peers could help students learn mathematics, and that their explanations would be used for this purpose. Participants were allowed 30 min to work on the packet. Based on pilot testing, this should have allowed students sufficient time to complete the problems.

We coded each response in the explanation phase for the presence or absence of three binary codes, each based on a priori ideas about how different ways of interacting with the problems might reflect different kinds of thinking. Describing the steps necessary to solve the problem was intended to address Skemp’s (1976) conception of an instrumental understanding and what Hiebert and Lefevre (1986) describe as procedural knowledge (i.e., “step-by-step [prescriptions for] how to complete tasks,” p. 6). Explaining mathematical relationships was intended to address Skemp’s conception of relational understanding and what several authors have referred to as conceptual knowledge (e.g., Baroody, 2003; Haapasalo, 2003; Hatano, 2003; Hiebert & Lefevre, 1986). Though not technically an “explanation,” we also coded attempts to solve the problem. This code was intended to address an (instrumental) belief that the goal of mathematics is to calculate (even if such a calculation is neither requested nor possible).

The three codes were not mutually exclusive, meaning that a student could produce more than one type of explanation in a single response. Each participant was given a summary score for each code. These scores ranged from 0 to 3, reflecting the number of problems on which the student’s explanation received the code.

Responses were coded positively for attempting to solve the problem if students produced a written record indicating any attempt at solving the problem. Because we were interested not in the correctness of their explanations but rather in whether students approached the task as if a numeric solution was expected, an explanation could be coded as attempting to solve the problem whether or not the solution was correct. Explanations were coded as describing steps if the student (1) described steps that could be part of a problem solution, or (2) wrote (and perhaps solved) an equation, but explained neither the values nor the operations in it. For example, “Add 22 and 20. Subtract that from 66,” “Subtract 6 from 38, then add 13. Then subtract 7 from your answer and add 4.” Common across all cases was that students approached the problem as a series of steps, without any explanation of the values used in a problem, how they related to one another, or why certain operations were necessary. Again, the steps they described did not need to be correct.

**Fig. 1:**
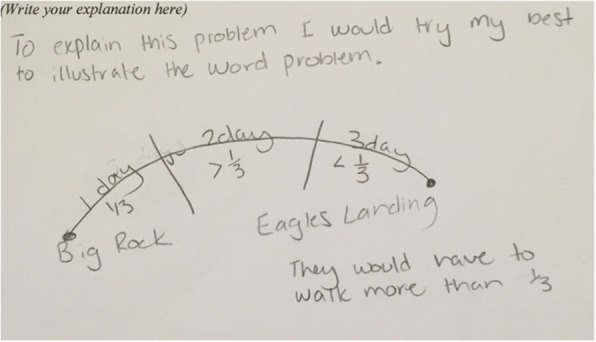
Student response that illustrated the relationship between values in the problem. Note: The student’s misunderstanding of the “less than” and “greater than” signs is irrelevant to the coding

Finally, explanations were coded as explaining mathematical relationships if the student described or illustrated a relationship between an element of the problem and a particular mathematical procedure, or between different quantities in the problem. For example, “You have to multiply by 2 so people can get their seconds.” See Fig. 1 for one student’s illustration of a relationship. Alternatively, the student may have provided an equation that included a rationale for at least one value or operation in it. Explanations coded as explaining mathematical relationships may also have referred to steps, as in, “You start by adding how much they walked on the first two days,” or, “Next you take the number of cupcakes you need and divide by how many cupcakes there are in a box.” But these were not coded as describing steps because the explanations went beyond the mere description of how to execute a computation. As with the other codes, explanations did not need to be correct.

Interrater agreement on the type of explanation(s) represented in each response exceeded 90%. Discrepancies were discussed until consensus was achieved.

### Results

#### Performance on the transfer test

The transfer test was analyzed in two ways. First, the total number correct across all four questions was calculated for each student, and means were compared across the two groups of students. Students in the non-calculable condition (mean (M) = 2.38, SD = 1.02) performed significantly better than those in the calculable condition (M = 1.50, SD = 1.37; *t*(30) = 2.05, *p* = .049, Cohen’s *d* = 1.10). Second, to isolate possible effects on the single, far transfer item, the chi-square test of independence was conducted comparing the frequency of correct responses on that item, across the two conditions. Although three times as many students in the non-calculable condition answered correctly compared to students in the calculable condition (6 of 16 versus 2 of 16), the difference was not statistically significant (*X*^2^(1) = 2.67, *p* = .103).

#### Explanations

To better understand students’ thinking as they worked on the three problems in the explain phase of the study, we conducted exploratory analyses of the explanations they produced. There was no observed difference between the two groups in the frequency with which they attempted to solve the problems, nor did the two groups did differ in the number of explanations they provided that described steps or explained mathematical relationships (see Table 1).

**Table 1:** Average number of explanations of each type by group (study 1)

Type of explanation	Calculable (*N* = 16) Mean (SD)	Non-calculable (*N* = 16) Mean (SD)	Group comparison
Attempted to solve	1.81 (1.28)	1.06 (1.06)	*t*(30) = 1.81 *p* = .081 Cohen’s *d* = 0.64
Describing steps	0.81 (0.75)	0.63 (0.81)	*t*(30) = 0.68 *p* = .501 Cohen’s *d* = 0.23
Explaining mathematical relationships	1.06 (1.00)	0.94 (1.00)	*t*(30) = 0.354 *p* = .726 Cohen’s *d* = 0.12

*Note.* Scores ranged from 0 to 3, with 3 meaning a student produced an explanation of that type for each of the three problems

Other, descriptive analyses suggest that participants in the non-calculable condition were particularly perplexed about how to respond to our request for explanations. Fifty percent of them stated explicitly in at least one of their responses that numbers were needed in order to answer our query. Forty-four percent made up numbers on at least one of the problems in order to solve and respond.

### Discussion

Our primary hypothesis, supported by the results of this study, was that removing students’ opportunities to calculate would improve their performance on the transfer test. Indeed, students asked to explain three problems that did not include numbers with which they might calculate an answer performed better on the transfer test than did students asked to explain problems that included numbers sufficient to calculate an answer.

Stacey and MacGregor (1999) discussed how patterns of thinking based on familiar routines can deflect students’ use of new, more sophisticated ways of thinking. Removing numbers with which to calculate was our attempt to deflect students from their reliance on calculation. Not being able to calculate, students had to come up with another approach, which might have caused them to spend more time considering what the problem asked and at least opened an opportunity for them to discover its underlying structure - more in line with the approach to problems taken by more skilled individuals (Chi et al., 1982; Hegarty et al., 1995; Hinsley et al., 1977; Larkin et al., 1980; Larkin & Simon, 1987; Lesgold, 1988; Mayer, 1985; Newell & Simon, 1972).

In our examination of students’ written work, we had hoped to find evidence of different representations of the problems across the two groups. However, we found no such differences. We cannot rule out the possibility that these null effects were due to the limited sample size, something we hoped to remedy in a replication study. What we did frequently see in explanations produced by the non-calculable group was evidence of a desire to have numbers at their disposal. Half of students expressed that desire explicitly, and more than half made up numbers so they could calculate an answer.

Although we demonstrated that students in the non-calculable condition performed significantly better on the transfer test than those in the calculable condition, we made little headway in understanding the mechanism underlying the effect. Perhaps a larger sample, and a richer written record of students’ work, would tell us more in this regard. This, and the desire to replicate our main finding, formed the motivation for our second study.

## Study 2

In study 2, we hoped to replicate the main finding from study 1, that removing students’ opportunities to calculate by omitting numbers would improve their performance on subsequent transfer problems. We hoped also to better understand the mechanism underlying the effect of removing numbers. To meet that objective, we added a stipulation to the instructions that students include a drawing in their responses. We expected that this addition would have multiple effects. First, requiring students to produce a drawing as part of their explanations might reinforce our request that they explain problems (rather than solve them). Second, requiring a drawing might encourage students to think more deeply about the structure of the problems. And finally, it might enrich the written record of students’ work.

The National Council of Teachers of Mathematics (NCTM) *Principles and standards for teaching mathematics* (2000), state that drawings and other informal representations serve as tools for thinking about problems and various studies have supported that claim (Clements & Battista, 1992; Goldin, 2002; Presmeg, 1986; Slovin, 2000; Zimmerman & Cunningham, 1991). Many of the structures that underlie textbook word problems are basic, quantitative relationships - for example, the part-whole schema or path schema, as identified by Lakoff (1990). These fundamental, underlying structures are able to be easily visualized in drawings. Encouraging students to produce drawings might help them tap into their understandings of these structures and apply them to the problem at hand. In younger students, for example, generating a graphic representation has been shown to help students “unpack” the structure of a word problem (Edens & Potter, 2008).

We made no demand with respect to when in their explanation students were to create a drawing, be it before or after they produced their verbal explanation. In line with the recommendations of other researchers (Presmeg, 1992; Zazkis, Dubinsky, & Dauterman, 1996), our aim was simply to encourage students to draw upon multiple ways of thinking about the problem. The items themselves were otherwise the same as in study 1.

In study 2 we also added the Single-Item Mathematics Anxiety scale (SIMA; Núñez-Peña, Guilera, & Suárez-Pellicioni, 2014). Mathematics anxiety has consistently been found to be inversely related to performance on mathematics tasks (Ashcraft, 2002; Ashcraft & Kirk, 2001; Hembree, 1990; see also meta analyses by Hembree, 1990 and Ma, 1999). Consistent with processing efficiency theory (Eysenck & Calvo, 1992), mathematics anxiety causes individuals to focus on intrusive thoughts and worries, thereby usurping the working memory that would have otherwise been available to apply to the task at hand (Ashcraft, 2002). We therefore expected anxiety to be inversely related to transfer test performance.

Particularly relevant to the current study is recent work by Ramirez and his colleagues (Ramirez, Chang, Maloney, Levine, & Beilock, 2016), who found mathematics anxiety to be negatively related to students’ use of more advanced problem-solving strategies. When anxiety was heightened, students appeared to retreat to strategies with which they were most familiar. Explaining problems and using drawings to do so offered students an opportunity to engage in more relational thinking. But because such activity is infrequent in mathematics classes and unfamiliar to students, we expected that students with high anxiety would be less likely to profit from this opportunity for sense-making than would students with low levels of anxiety. Highly anxious students might opt to stick with immediate calculation because it is a familiar strategy. Students with low anxiety, in contrast, might be more likely to take advantage of the opportunities for sensemaking presented by our removal of numbers from the problems.

### Method

#### Participants

Eighty-one students enrolled in Elementary Algebra courses participated in study 2. They were enrolled at a different Southern California community college than were participants in study 1. Students were distributed almost evenly across four intact classes (two sections taught by each of two instructors). Data collection took place during class time and although participation was voluntary, no students declined to participate. Participants included 37 female, 38 male, and 1 transgender student, and 5 students who declined to state their sex. They varied in age from 17 to 41 years (M = 22, SD = 4.76). More than half of students (51%) were of Hispanic or Latino origin, 22% were white, 6% Asian, 6% black or African American, 5% were of mixed ethnicity, 3% were American Indian, Alaska native, native Hawaiian or other Pacific Islander, and the remainder (7%) declined to state their ethnicity.

We did not conduct a power analysis prior to designing study 2; due to the study context we would not have been able to increase our sample size beyond the 40 in each group in any case. However, a post-hoc power analysis (Rosner, 2011) based on the pooled observed standard deviations from study 1 showed power of 85% for detecting a 0.75-point difference on the transfer test with 40 students per group (pooled SD = 1.12, *p* < .05), and 92.8% for detecting an effect of 0.75 points on the type of explanation variables (pooled SD = 0.98, *p* < .05).

#### Materials

The same materials were used in study 2 as in study 1, with two exceptions. As in study 1, there were two versions of the materials (calculable and non-calculable), each containing the same seven mathematics problems as used in study 1. As in study 1, students were asked to write explanations for the first three problems, and then asked to solve the final four problems. Different from study 1, the instructions this time included a request that students produce a drawing as part of each explanation. Instructions were repeated for each of the three problems, which we hoped would increase the likelihood of students including drawings in their explanations.

Specifically, the instructions said, “We are going to present you with three mathematics problems. Study each problem until you are sure that you understand it, then write an explanation that you think would help another student understand the problem. Use a drawing as part of your explanation.” Each question was followed by a box in which students could show their work, and in the box was written, “Write your explanation here.” The final four problems, which constituted the transfer test, were the same as in study 1.

The materials for study 2 differed from those of study 1 in one other way. In study 2, the packet that participants completed included a final page with demographic questions and also the question, “On a scale from 1 to 10, how mathematics anxious are you?” The anchors for the scale were 1 (not anxious) and 10 (very anxious).

#### Experimental design and procedure

As in study 1, individual participants in each class were randomly assigned to receive one of the two versions of the materials, resulting in 40 participants in the calculable group and 41 in the non-calculable group. Materials were distributed at the beginning of a regular class period and participants were given 30 min to work on the materials. They were asked not to return to prior questions once they had advanced through the packet.

### Results

#### Performance on the transfer test

Students’ performance on the transfer test differed significantly across conditions. Replicating the result in study 1, students in the non-calculable condition (M = 2.00, SD = 1.18) outscored students in the calculable condition (M = 1.25, SD = 1.03) on the four transfer items (scored from 0 to 4 correct; *t*(79) = 3.04, *p* = .003; Cohen’s *d* = 0.68). Also, more participants in the non-calculable group (10 of 41) got the single far transfer item correct than did participants in the calculable group (3 of 40; *X*^2^(1) = 4.287, *p* = .038).

#### Students’ explanations

As in study 1, we conducted analyses of the explanations that students produced for the first three problems. As in study 1, participants in the calculable group were more likely to attempt to solve the problems than were participants in the non-calculable group (see Table 2). The fact that the problems they explained had numbers, and thus were calculable, is surely a central reason for their providing more numerical answers. However, participants in the non-calculable group also provided numeric solutions, even though they had to take numbers out of thin air in order to produce them. One participant in the non-calculable group, for example, answered problem 3 with, “Depending on how many of Andrew’s friends were coming to this party, I’d say buy a dozen boxes.”

**Table 2:** Average number of explanations of each type by group (study 2)

Type of explanation	Calculable *(N* = 40) Mean (SD)	Non-calculable *(N* = 41) Mean (SD)	Group comparison
Attempted to solve	2.53 (0.82)	1.44 (1.16)	*t*(79) = 4.85 *p* < .0001 Cohen’s *d* = 1.09
Describing steps	1.00 (1.09)	0.32 (0.69)	*t*(79) = 3.39 *p* = .001 Cohen’s *d* = 0.75
Explaining mathematical relationships	1.40 (1.19)	1.05 (0.95)	*t*(79) = 1.47 *p* = .146 Cohen’s *d* = 0.33

Note, scores ranged from 0 to 3, with 3 meaning a student produced an explanation of that type for each of the three problems

Students in the calculable group also more frequently described steps than did students in the calculable group. The average number of times students explained mathematical relationships did not differ significantly across groups. Students, on average, explained relationships on barely more than one of the three problems (see Table 2).

Twenty-nine percent of students in the non-calculable group stated explicitly in at least one of their responses that numbers were needed in order to answer. Nearly half of the students in the non-calculable group (44%) at least once made up numbers in order to produce a numerical answer.

Despite our clear instructions, many students never produced a drawing as part of their explanations (50% in the calculable group, and 24% in the non-calculable group). And, the average number of drawings produced by students in the calculable group (M = 1.08, SD = 1.25 did not differ from the average number of drawings produced by students in the non-calculable group (*t*(79) = 1.58, *p* = .118, Cohen’s *d* = 0.35).

Drawings were coded as either useful or not useful. The need for this code arose from the data. Our aim with requiring students to produce a drawing as part of their explanation was that doing so might help them discover the underlying structure of the word problem. However, many of the drawings that the students produced were no more than illustrations of the problem scenario. Non useful drawings included things such as illustrations of the mountains in which the hike in problem 1 took place. Though this drawing may have helped students imagine the setting of the problem, it did not include any representation of the mathematical relationships.

We defined drawings as useful, on the other hand, if they had the potential to support reasoning about the mathematical relationships in the problem. Useful drawings included things such as a line segment that represented the total distance of the hike in problem 1, divided into segments to represent the distance traveled on each of the 3 days. Interrater agreement on the usefulness of drawings exceeded 97%. Discrepancies were discussed until consensus was achieved. The calculable and non-calculable groups produced roughly the same number of useful drawings (M = 0.75, SD = 0.75 vs. M = 0.76, SD = 0.89, respectively; *t*(79) = 0.03, *p* = .976, Cohen’s *d* = 0.01).

There was no significant correlation between the provision of a drawing and transfer test performance (*r* = 0.03, *p* = .778), nor when the analysis was limited to useful drawings (*r* = −.00, *p* = .966).

#### Comparing students’ explanations across the two studies

**Fig. 2:**
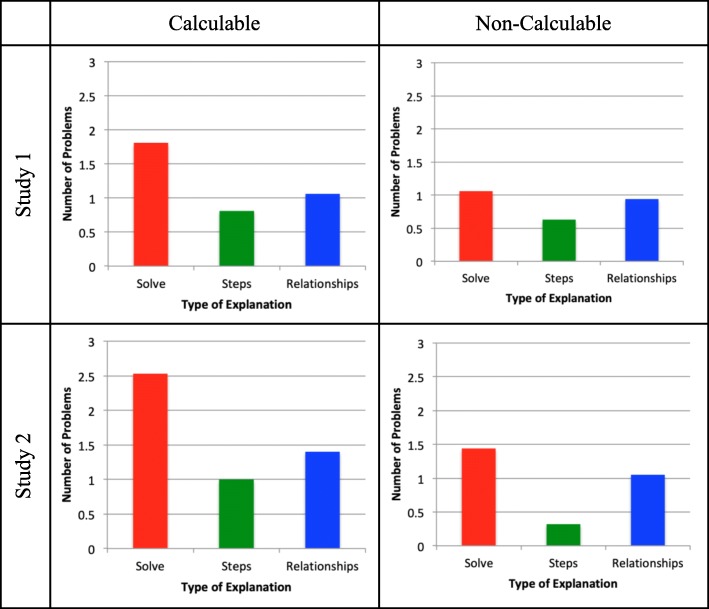
Number of problems students “explained” in different ways, across studies and conditions

Our goal in examining students’ explanations was to gain insight into what students were thinking as they engaged in the explanation task (see Fig. 2 for a comparison of the results of the two studies). Mixed analysis of variance (ANOVA) was run separately for each of the two studies with experimental condition as a between-subjects variable (calculable, non-calculable) and explanation type, a within-subjects variable (solved, described steps, explained mathematical relationships). In study 1 there was no significant interaction between experimental condition and explanation type (*F*(2, 60) = 0.97, *p* = .383) nor a main effect of experimental condition (*F*(1, 30) = 2.91, *p* = .099). There was, however, a significant main effect for explanation type (*F*(2, 60) = 4.32, *p* = .018). Post hoc comparisons using the Tukey honestly significant difference (HSD) test indicated that the mean score for attempting to solve (M = 1.44, SD = 1.22) was significantly higher than the mean score for describing steps (M = 0.72, SD = 0.77; p = .015). Neither differed from explaining mathematical relationships (M = 1.00, SD = 0.98; *p* = .197 and *p* = .506, respectively).

In study 2, as in study 1, there was no significant interaction between experimental condition and explanation type (*F*(2, 158) = 2.64, *p* = .075). However, there was a significant main effect of experimental condition (*F*(1, 79) = 33.10, *p* < .001) and of explanation type (*F*(2, 158) = 34.26, *p* < .001). Students in the calculable condition produced more explanations overall (M = 4.93, SD = 1.61) than did students in the non-calculable condition (M = 2.80, SD = 1.71), perhaps indicating that students were confused about what to do when the numbers were not there. Post hoc comparisons using the Tukey HSD test indicated that the mean score for attempting to solve (M = 1.98, SD = 1.14) was significantly higher than the mean score for explaining mathematical relationships (M = 1.22, SD = 1.08; *p* < .0001), which was in turn higher than the mean score for describing steps (M = 0.65, SD = 0.96; *p* = .002).

#### Mathematics anxiety

Participants reported moderate levels of mathematics anxiety (M = 6.45, SD = 2.67), with levels in the calculable condition (M = 6.20, SD = 2.68) that were not statistically different from those in the non-calculable condition (M = 6.72, SD = 2.66; *t*(77) = 0.88, *p* = .382, Cohen’s *d* = 0.20). Responses in each condition reflected the full range of the anxiety scale. Anxiety was negatively correlated with transfer test performance (*r* = −.400, *p* < .001) across the sample as a whole, meaning that students with higher anxiety scored lower on the transfer test than did students with lower anxiety. Anxiety was negatively associated with transfer test performance within the non-calculable group (*r* = −.505, *p* < .001), but not within the calculable group (*r* = −.251, *p* = .128). Within-group correlations were not significantly different from each other (*Z* = 1.3, *p* = .097). In neither condition was anxiety related to the frequency with which students described steps or explained mathematical relationships. However, within the non-calculable group, students with higher mathematics anxiety were significantly more likely to make up numbers so they could produce numerical answers than were students with low anxiety (*r* = .325, *p* = .038).

### Discussion

Study 2 sought to replicate the primary finding of study 1, that omitting numbers from problems that students are asked only to explain improved their ability to solve subsequent transfer problems. The finding was replicated, not only for the 4-item transfer test but also for the single far transfer item when analyzed separately. In preventing students’ ability to calculate, we may have freed them up to explore more sophisticated practices (Stacey & MacGregor, 1999), and they used those new understandings on subsequent problems.

In examining what students wrote when they were asked to explain problems, we found that students in the calculable group more frequently attempted to solve the problems, and more frequently described the steps to solve them, than did students in the non-calculable group. Both of these coding categories reflect an instrumental view of math (Skemp, 1976), suggesting that our removing numbers from problems might have moved students in the non-calculable group away from this view. However, although we would have expected a concomitant shift toward a relational approach, we did not see evidence for this in the data. Students in the non-calculable group explained relationships no more frequently than did students in the calculable group.

Based on the main effect for experimental condition in study 2, removing numbers from problems appears to have caused a general suppression of students’ written explanations as indicated by the lower overall frequency with which the explanation codes were applied. Thus, the better transfer performance of the non-calculable group may have resulted from just the extra effort required to figure out what was being asked on these problems without numbers. Students in the non-calculable group, similar to expert problem solvers, may just have allocated more of their time to trying to understand the problem (Hegarty et al., 1995; Lesgold, 1988). Even the act of concocting numbers when none were provided might have led to deeper thinking than did calculating with the numbers that had been provided.

In study 2, we added the request that students provide a drawing as part of each of their explanations. Participants in the calculable and the non-calculable groups provided drawings at the same rate. But overall rates of including drawings were low and uncorrelated with transfer test performance. If the drawings served as tools for thinking more deeply about the problems and, in particular, more deeply about their structure (Clements & Battista, 1992; Edens & Potter, 2008; Goldin, 2002; Lakoff, 1990; Presmeg, 1986; Slovin, 2000; Zazkis et al., 1996; Zimmerman & Cunningham, 1991), our coding scheme did not capture it.

Self-ratings of mathematics anxiety were similar across conditions and, as expected, inversely related to transfer test performance. Interestingly, within the non-calculable group, anxiety was positively correlated with making up numbers with which to calculate. This finding is consistent with work suggesting that anxious students fail to use advanced problem-solving strategies, opting instead for familiar ones (Ramirez et al., 2016).

## General Discussion

In the two studies reported here, we explored whether removing opportunities to calculate could improve students’ subsequent ability to solve similar word problems. Our hypothesis that it might do so was grounded in Skemp’s (1976) theory that there are two forms of mathematical understanding: instrumental understanding (rules without reasons) and relational understanding (knowing what to do and why). The former offers more limited rewards (e.g., quick answers) whereas the latter offers longer-term benefits (e.g., more flexible thinking). By removing students’ opportunities to calculate, we removed their ability to exercise instrumental thinking, which we hoped would move them toward more relational thinking, and ultimately to more transferable knowledge. We found some support for this hypothesized chain of events in the non-calculable group’s superior performance on the transfer test, which was replicated across the two studies.

Evidence for the mechanism behind the improvement was less clear. A lack of numbers with which to calculate led to a lower frequency of explanations incorporating strategies that were instrumental in nature, but it also led to a lower frequency of incorporating strategies that were relational in nature. Taking away numbers appeared to suppress explanations altogether - at least as defined by our coding categories. Further, it seems that we were unable to suppress students’ desire to use instrumental strategies. Many of the students in the non-calculable group wanted so much to be able to calculate that they at least once made up numbers so that they could perform the calculations for which they yearned. Although we may have removed the immediate opportunity to calculate, these students found a way to re-introduce it. This behavior was positively associated with students’ level of mathematics anxiety. Consistent with prior research (Ramirez et al., 2016), individuals with high anxiety found instrumental thinking particularly desirable.

Admittedly, explaining a problem, whether there are numbers in it or not, is a novel task for most students. Math teaching in the USA, from kindergarten through community college, is filled with the execution of procedures (Grubb and Associates, 1999; Stigler & Hiebert, 1999). The task was particularly difficult for students who were asked to provide an explanation when they had not calculated an answer, as this is a task in which likely very few had experience. Students across both conditions might have solved the problem, instead of explaining it, because they do not know how to do anything other than compute. Or, maybe they did not bother to read the instructions; they know that when there is mathematics to do, there are computations to produce. Or, perhaps they thought that the experimenter could not possibly have meant for them to omit a computation and its result.

Given the group differences we found on the transfer test though, following our instructions *successfully* appears to be less important than *attempting* to follow the instructions. Struggling to understand what was being requested - struggling to produce any kind of explanation of the problem - might have by itself been a productive task (see struggle in Hiebert & Grouws, 2007), and sufficient to make students think more deeply about the problems. It could be that the ability to produce a particular kind of explanation (or even any explanation at all) is not necessary in order to disrupt instrumental thinking. Simply slowing down students’ ability to calculate an answer might afford new opportunities for thinking and learning - a finding with clear implications for classroom teaching.

A final question to consider is what, exactly, students in the non-calculable group learned that enabled them to outperform their peers on the transfer test. Did they mainly learn how to spot the specific structure that was embodied in the three problems they explained or were they additionally nudged into adopting more of a thinking and sensemaking frame of mind? Performance on the far transfer item suggests an answer. In study 2, the non-calculable group significantly outperformed the calculable group on this item. Across both studies, the number of students answering the far transfer question correctly in the non-calculable group was three times as great as the number of students in the calculable group. Though a single item is hardly sufficient to draw a firm conclusion, it does point to the possibility that students became more inclined to apply sensemaking in general to a problem that differed in structure from the ones they had just practiced explaining.

## Limitations and proposals for further research

A few limitations of our work bear mention. First among them is the small sample size in our first study. Our null results may have been caused by insufficient power to detect group differences. Second is the number of problems on the transfer test. A more robust measure of transfer would require a larger number and wider variety of problems. Third is the nature of the problems in our manipulation. Removing numbers from the problems may have resulted in an imperfect alignment between the original and the altered item. In particular, one of the problems in the non-calculable condition included a fraction, whereas the comparable problem in the calculable condition did not. The fraction might have benefited students by prompting them to think proportionally, but it also might have harmed students’ thinking by increasing their anxiety. The whole number, on the other hand, might have led students to focus on the sequential nature of the problem, thereby increasing the likelihood that their solution focused on steps. Each of these limitations should be addressed in future research.

Other questions warrant exploration as well. The group differences we saw in transfer test performance resulted from a very brief experimental manipulation. Being pressed to make sense of just three problems made a measurable - though we assume temporary - impact on students. What remains to be seen is how long the impact lasts, how far it generalizes to different kinds of mathematics problems, and how it might be strengthened by more extended opportunities for sensemaking.

Future research designs should manipulate the dosage of students’ exposure to non-calculable problems, especially in combination with delayed post-tests, so that we might better understand how the amount of exposure is related to the lastingness of its effects. Future studies might also employ more complex mathematical tasks. It might not be possible to remove numbers from a more complex problem and still end up with a reasonable non-calculable problem, but one can imagine teaching more advanced mathematical concepts by reducing instrumental thinking in some other way. The key might not be our particular method but rather that we found a way to increase the likelihood that students would try to make sense of a mathematical problem. Studies of all varieties would profit from the inclusion of interviews with students to illuminate how they approach problem solving both before and after the intervention. And transfer test problem sets should be expanded to include multiple problems that differ in structure from those used in the intervention, so that we might better understand the extent to which students transfer a different way of thinking to new problems.

Although we are far from understanding the mechanism that produces the effect, we are confident that there is indeed an effect worth explaining. This small experimental finding suggests to us that interventions designed to help students remediate prior mathematical failure should perhaps focus less on the specific skills students are lacking, and more on the dispositions they bring to the task of “doing mathematics.”

## Data Availability

The datasets used and/or analyzed during the current study are available from the corresponding author on reasonable request.

## Appendix

Mathematics problems for which participants were asked to write an explanation (i.e., problems in the experimental manipulation).

**Table:**

Calculable condition	Non-calculable condition
A group of tourists planned a 3-day walking trip from Big Rock to Eagles Landing, a total of 66 km. On the first day they walked 22 km. On the second day they walked 20 km. How far would they have to walk on the third day of their trip?	A group of tourists planned a 3-day walking trip from Big Rock to Eagles Landing. On the first day they walked one third of the total distance. On the second day they walked a little less. How far would they have to walk on the third day of their trip?
A bus left the station and headed downtown. At the first stop, 7 people got off the bus and 4 people got on. At the second stop, 6 people got off and 13 got on. After the second stop there were 38 people on the bus. How many people were on the bus before it made its first stop?	A bus left the station and headed downtown. At the first stop, some people got off the bus, but only a few people got on. At the second stop, a few people got off, but even more got on. After the second stop the bus was full. How many people were on the bus before it made its first stop?
Andrew was planning a party for 20 people. He found a bakery that had amazing cupcakes, and wanted to make sure each person could have at least 2. The bakery only sells the cupcakes in boxes of 6. How many boxes does he need to buy?	Andrew was planning a big party for his friends. He found a bakery that had amazing cupcakes, and wanted to make sure each person could have seconds. The bakery only sells the cupcakes in boxes of six. How many boxes does he need to buy?

The mathematics problems that participants were asked to solve (i.e., transfer test questions) are as follows:

1. Alisa started a YouTube channel with some followers on Friday. On Saturday 8 people unsubscribed her channel but 12 new people started following her. On Sunday 7 more people unsubscribed but 5 new people started following her channel which then had a total of 32 followers. How many followers did she start with on her channel on Friday?
2. Mark rented a 900-page book in the library. In the first day he read 225 pages, and on the second day he read 23 pages more than he did on the first day. How many pages does he have left to read to finish the book?
3. A boat has a maximum capacity of 80 passengers. The captain already greeted 8 newly married couples, 7 families of four, and 5 families of three. How many more people can board to reach the maximum number of people allowed on the boat?
4. There are 32 students in class – 23 of the students like cats, 18 like dogs, and 10 like both cats and dogs. How many students do not like either cats or dogs? [far transfer item]

## Footnotes

1 Community colleges are open access, 2-year schools that provide affordable postsecondary education. Programs normally culminate with an Associate’s degree and may serve as a pathway to a 4-year college or university.
